# Tuning the Transcriptional Response to Hypoxia by Inhibiting Hypoxia-inducible Factor (HIF) Prolyl and Asparaginyl Hydroxylases*[Fn FN2]

**DOI:** 10.1074/jbc.M116.749291

**Published:** 2016-08-08

**Authors:** Mun Chiang Chan, Nicholas E. Ilott, Johannes Schödel, David Sims, Anthony Tumber, Kerstin Lippl, David R. Mole, Christopher W. Pugh, Peter J. Ratcliffe, Chris P. Ponting, Christopher J. Schofield

**Affiliations:** From the ‡ Chemistry Research Laboratory, Department of Chemistry, University of Oxford, Oxford OX1 3TA,; the § Centre for Cellular and Molecular Physiology, University of Oxford, Oxford OX3 7BN,; the ¶ Computational Genomics Analysis and Training Programme, MRC Functional Genomics Unit Department of Physiology, Anatomy and Genetics, University of Oxford, South Parks Road, Oxford OX1 3PT, and; the ‖ Target Discovery Institute, University of Oxford, Oxford OX3 7FZ, United Kingdom

**Keywords:** erythropoiesis, hydroxylase, hypoxia, hypoxia-inducible factor (HIF), inhibitor, 2-oxoglutarate and ferrous iron dioxygenase, anaemia, metallo-enzyme inhibitor, oxygen sensing, transcriptional regulation

## Abstract

The hypoxia-inducible factor (HIF) system orchestrates cellular responses to hypoxia in animals. HIF is an α/β-heterodimeric transcription factor that regulates the expression of hundreds of genes in a tissue context-dependent manner. The major hypoxia-sensing component of the HIF system involves oxygen-dependent catalysis by the HIF hydroxylases; in humans there are three HIF prolyl hydroxylases (PHD1–3) and an asparaginyl hydroxylase (factor-inhibiting HIF (FIH)). PHD catalysis regulates HIFα levels, and FIH catalysis regulates HIF activity. How differences in HIFα hydroxylation status relate to variations in the induction of specific HIF target gene transcription is unknown. We report studies using small molecule HIF hydroxylase inhibitors that investigate the extent to which HIF target gene expression is induced by PHD or FIH inhibition. The results reveal substantial differences in the role of prolyl and asparaginyl hydroxylation in regulating hypoxia-responsive genes in cells. PHD inhibitors with different structural scaffolds behave similarly. Under the tested conditions, a broad-spectrum 2-oxoglutarate dioxygenase inhibitor is a better mimic of the overall transcriptional response to hypoxia than the selective PHD inhibitors, consistent with an important role for FIH in the hypoxic transcriptional response. Indeed, combined application of selective PHD and FIH inhibitors resulted in the transcriptional induction of a subset of genes not fully responsive to PHD inhibition alone. Thus, for the therapeutic regulation of HIF target genes, it is important to consider both PHD and FIH activity, and in the case of some sets of target genes, simultaneous inhibition of the PHDs and FIH catalysis may be preferable.

## Introduction

**FIGURE 1.**
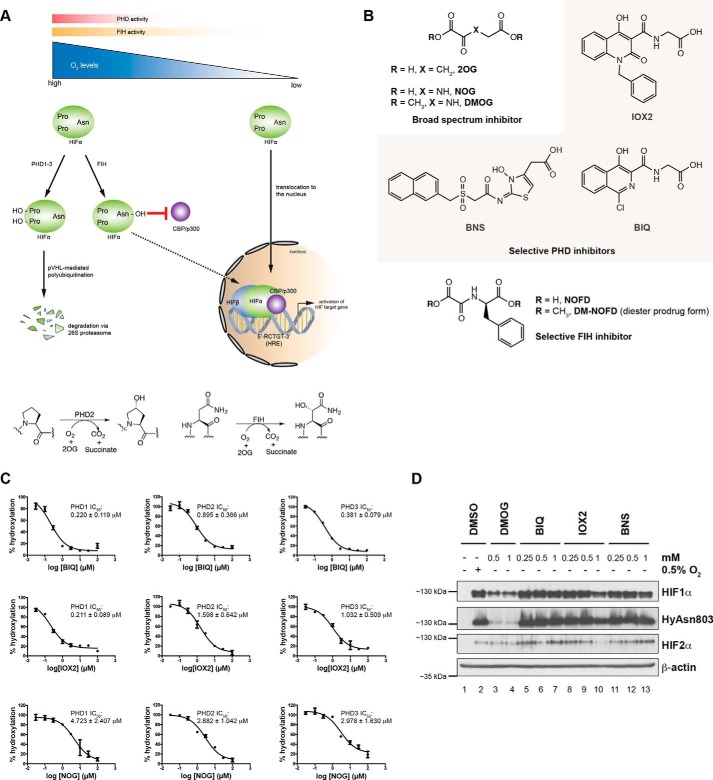
**Up-regulation of HIFα isoforms by the HIF hydroxylases.** *A,* hypoxia HIF-sensing pathway, showing the role of the HIF prolyl hydroxylases (PHDs) and asparaginyl hydroxylases (FIH). Prolyl hydroxylation occurs at either or both of the N- or C-terminal oxygen degradation domains (NODD or CODD). Each PHD/FIH-catalyzed reaction is coupled to the conversion of 2OG and O_2_ into succinate and carbon dioxide (CO_2_). *B,* chemical structures of IOX2, BNS, and BIQ (collectively referred to as the PHIs) and DM-NOFD (FIH inhibitor) used in this work. *C,* inhibition curves for BIQ (*top*), IOX2 (*middle*), and NOG (*bottom*) and their respective IC_50_ values as determined from *in vitro* hydroxylation assays for recombinant PHD1 (*left*), PHD2 (*middle*), and PHD3 (*right*). Each data point represents average signal ± S.D. (*n* = 3). *D,* immunoblots showing up-regulation of HIF1α and HIF2α proteins in MCF-7 cells after 16 h of treatment with the inhibitors, in comparison with hypoxic treatment (0.5% O_2_). Note the lack of inhibition of HIF1α asparaginyl hydroxylation by the more selective HIF PHIs, in comparison with the broad-spectrum 2OG-oxygenase inhibitor DMOG. For experimental details, see under “Experimental Procedures.” *NOFD, N*-oxalyl-d-phenylalanine; *DMSO,* dimethyl sulfoxide.

In animals, the cellular response to hypoxia, *i.e.* limiting oxygen availability, is predominantly orchestrated by the hypoxia-inducible transcription factors (HIFs)^6^ that work over a time course of hours to days to adapt cells and tissues to limiting oxygen availability (1). The α/β-heterodimeric HIF transcription factors can directly up-regulate hundreds of genes, including those encoding for erythropoietin (*EPO*) and vascular endothelial growth factor (VEGF) (1). Thus, therapeutic manipulation of the HIF system has substantial medicinal potential, *e.g.* by promoting EPO production for the treatment of anemia (2) or by down-regulating VEGF production in tumors (3). To date, the only validated cellular oxygen-sensing mechanism in humans for the HIF hypoxia-sensing system is provided by a set of 2-oxoglutarate (2OG) and ferrous iron-dependent dioxygenases. The three human isoforms of the HIF prolyl hydroxylase (PHD1–3) signal for HIFα degradation by catalyzing the *trans*-4-prolyl hydroxylation of HIF1α and HIF2α isoforms at either, or both, of two specific sites in the N- or C-terminal oxygen-dependent degradation domains (NODD and CODD, respectively) (4–8). Increases in HIF1α and HIF2α levels are associated with the up-regulation of different sets of HIF target genes; for example, HIF1α is principally associated with glycolytic gene (such as *PGK1*, *HK2,* and *LDHA*) up-regulation (9) and HIF2α with *EPO* up-regulation (10, 11). HIFα prolyl hydroxylation in the NODD and CODD regions serves as the recognition signal for the von Hippel Lindau protein, the targeting component of an ubiquitin E3 ligase complex (6–8, 12). Thus, HIFα isoforms are degraded in an oxygen-dependent manner by the ubiquitin-proteasome system. A second type of HIFα oxidation (asparaginyl hydroxylation) occurs in the C-terminal transcriptional activation domain (CTAD) of HIFα isoforms, as catalyzed by factor-inhibiting HIF (FIH); this hydroxylation blocks the interaction between HIFα and the p300/CBP family of transcriptional co-activator proteins (13–18). The sensitivity of HIF hydroxylase catalysis to oxygen availability is directly coupled to the stabilization and activation of HIF. Thus, the HIF hydroxylases act as cellular oxygen sensors with the PHD catalysis regulating HIF levels and FIH catalysis regulating HIF activity (Fig. 1*A*).

The HIF system regulates the expression of many genes by directly up-regulating their transcription (19, 20) and has the potential to indirectly regulate many other genes. The HIF system is therefore capable of profound cellular reprogramming. 2OG dioxygenases, such as the PHDs and FIH, are amenable to inhibition by small molecules, and PHIs are currently in advanced stages of clinical studies for the treatment of anemia through the HIF-mediated up-regulation of EPO (2, 21–23). The success of such inhibitors in part will likely be dependent on their ability to selectively up-regulate *EPO* gene expression in a sufficiently safe manner, *i.e.* with low toxicity and side effects. The extent to which selective transcription of *EPO* (for example) can be achieved and whether or not the concurrent up-regulation of the other HIF target genes is therapeutically desirable remains to be determined.

Distinct sets of HIF target genes are expressed in different cells/tissues, in a context-dependent manner (24). The mechanism(s) by which context-dependent HIF regulation of expression is achieved are of major clinical and basic scientific interest. In the latter case, this question is applicable to any pleiotropic transcriptional regulation system. Understanding and exploiting the chemical details of such context-dependent regulation of expression is a major challenge in contemporary molecular biology. Because of the strong induction of active HIFα isoforms in response to changes in atmospheric oxygen availability (hypoxia), it may be that the HIF system is a particularly good model for addressing such questions.

Although the precise regulatory mechanisms underlying the regulation of specific sets of HIF target genes are likely extremely complex from a chemical perspective (*e.g.* involving the combinatorial modifications on the histone H3 N-terminal tail), some such mechanisms are likely to be more important than others, at least in terms of the physiological hypoxic response. In this regard, the differential roles of the PHDs and FIH are of particular interest; the available evidence is that PHD activity is more sensitive to hypoxia than that of FIH, as supported by studies with both cellular and isolated enzymes (25–27). Moreover, there are few studies on how the PHDs and FIH might differentially affect transcription of specific genes (28–31). Such studies are of interest with regard to the therapeutic manipulation of HIF target genes, *i.e.* “dual action” PHD and FIH inhibition may be desirable in some, but not other, cases. More generally, there is the question of how well small molecules targeting the HIF hydroxylases mimic physiological hypoxia.

Here, we report studies investigating the extent to which HIF target gene expression is regulated by the PHDs and FIH. Our results imply that the role of FIH in regulating HIF-responsive gene expression varies substantially, both in terms of the HIF target genes in the same cell and the same HIF target genes in different cells. The results also revealed the unexpected result that broad-spectrum 2OG dioxygenase inhibitor is a better mimic of the transcriptional response to hypoxia than selective PHD inhibitors, at least in the studied cell line.

## Results

### 

#### 

##### DMOG Better Mimics the Transcriptional Response to Hypoxia than Selective PHD Inhibitors in MCF-7 Cells

We used high throughput RNA sequencing (RNA-seq) to investigate the cellular transcriptional response to hypoxia as well as the effects of three small molecule inhibitors that have been reported to be selective (at least over some, but likely not all, other human 2OG oxygenases) for the PHDs (FG2216/BIQ, IOX2, and BNS; collectively referred to as the PHIs) or a broad-spectrum 2OG analogue, dimethyloxalylglycine (DMOG) (Fig. 1*B*). DMOG is a prodrug form of *N*-oxalylglycine (NOG), which has been extensively used as a 2OG dioxygenase inhibitor in cellular and animal studies (27, 32, 33). The three PHD inhibitors were selected because one of them has been used in a clinical trial of anemia (BIQ/FG2216) (34), one has been profiled in some detail for selectivity and potency (IOX2) (35, 36), and another (BNS) has a substantially different heteroaromatic structure (37). *In vitro* hydroxylation assays for PHD1–3 indicate that the PHIs (BIQ and IOX2) and NOG potently inhibit all three of the human PHDs (Fig. 1*C*). In our cell-based studies, we tested human breast cancer MCF-7 cells treated under normoxia, hypoxia (0.5% O_2_), or with the small molecule inhibitors (DMOG, IOX2, BNS, and BIQ). MCF-7 cells were selected in part because they are known to up-regulate both HIF1α and HIF2α in response to hypoxia (38). “Optimal” concentrations of the small molecule inhibitors required for the induction of both HIF1α and HIF2α (to approximately the same level detected under 0.5% O_2_) were first determined by immunoblotting (Fig. 1*D*). We then treated the MCF-7 cells with the experimentally determined concentrations of 250 μm IOX2, 500 μm BIQ, 250 μm BNS, 1 mm DMOG or 0.5% O_2_ for 16 h before profiling for genome-wide gene expression changes using RNA-seq (*n* = 2 per condition).

Differential expression analysis confirmed clear hypoxia-induced changes in our RNA-seq data set with the transcription of 1081 genes identified as being up-regulated in hypoxia compared with normoxia (supplemental data). This set of genes was enriched for KEGG pathways known to be involved in the cellular response to hypoxia, including glycolysis/gluconeogenesis (hypergeometric test; fold enrichment = 5.22, FDR = 1 × 10^−6^). Furthermore, we reproduced the induction of genes proposed to reflect a core hypoxic “signature” (39), namely *ADM*, *AK3L1*, *BNIP3*, *CA9*, *CCNG*, *ENO1*, *HK2*, *LDHA*, *PFKFB3*, *PGK1*, *SLC2A1,* and *VEGFA* (all with fold changes >2 and FDR <0.05).

**FIGURE 2.**
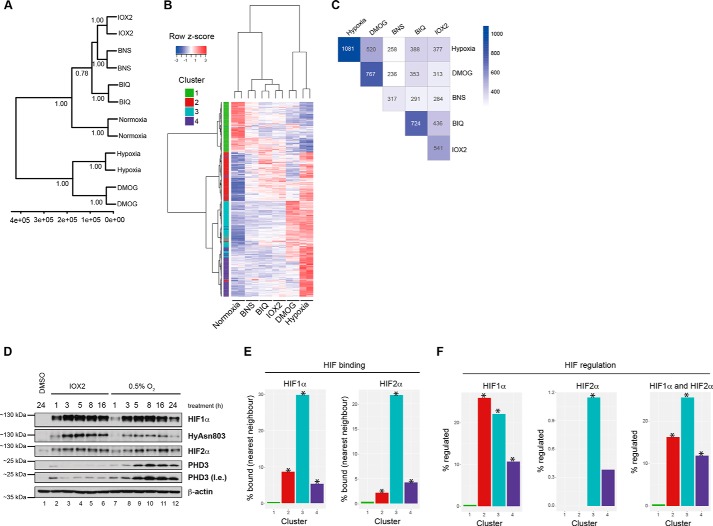
**DMOG better mimics the transcriptional response to hypoxia than selective PHI in MCF7 cells.** *A,* hierarchical clustering (Manhattan distance, Ward's linkage) of samples based on FPKM values of 13,351 genes. Clustering was performed using the pvclust package in R-3.1.3. The numbers represent the bootstrap probabilities (*BP*) based on 1000 bootstrap resamples. Values of >95% represent highly supported clusters. *B,* hierarchical clustering (Manhattan distance, Ward's linkage) of genes and samples based on FPKM values of 1081 hypoxia-regulated genes. The *left panel* of the heatmap represents cluster assignments of genes based on *k* means clustering with *k* = 4 using the *k* means function in R (version 3.1.3). *C,* overlap of genes that were called as differentially regulated between each experimental condition and normoxia. *D,* immunoblots showing the time course of HIFα induction in MCF-7 cells by IOX2, one of the selective PHIs in comparison with hypoxic treatment. *E,* overlap of HIF1α and HIF2α binding and gene clusters. A published list of genes annotated as nearest neighbors to HIF-binding sites (40) was intersected with each cluster of genes from RNA-seq analysis. The proportion overlapping was plotted. Significance of the overlap was determined using a sampling procedure described under “Experimental Procedures.” *F,* overlap of HIF1α and HIF2α-regulated genes by siRNA. Publicly available RNA-seq data for siRNAs against HIF1α, HIF2α, or both were analyzed and the overlap between genes down-regulated upon knockdown. *DMSO,* dimethyl sulfoxide; *l.e.,* long exposure. *, *p* < 0.01.

We then investigated the extent to which the PHIs mimicked the transcriptional response to hypoxia. Overall, the different types of selective PHIs all displayed similar transcriptional profiles to each other; these were clearly distinct from those observed for normoxia (Fig. 2*A*). Notably, we observed that the transcriptional response to the broad-spectrum inhibitor DMOG was more similar to the hypoxic response than it was for any of the selective PHI (Fig. 2*A*). These observations were supported by more studies in which we restricted the analyses to those genes that were regulated by hypoxia (Fig. 2*B*). Hierarchical clustering of hypoxia-regulated genes revealed the presence of four clusters (Fig. 2*B*); we assigned each gene to one of four clusters using *k* means clustering (*k* = 4). These clusters represent the following: those genes that were down-regulated in hypoxia and DMOG, but to a lesser extent by the PHI (cluster 1); those that were up-regulated by hypoxia, DMOG, and the PHI (albeit to a varying degree) (cluster 2); those that were up-regulated by hypoxia and DMOG, but to a lesser extent by the PHI (cluster 3); and those that were only up-regulated by hypoxia (cluster 4). The identification of clusters that represent genes regulated predominantly by hypoxia and DMOG as opposed to the PHI is manifested as a greater overlap of differentially expressed genes between these conditions when compared with normoxia (Fig. 2*C*). Indeed, DMOG regulated ∼50% of hypoxia-regulated genes compared with ∼35, ∼35, and ∼25% for BIQ, IOX2 and BNS, respectively. This pattern is not due, at least solely, to temporal or magnitude differences in the induction of HIF1α/HIF2α between hypoxia and PHI, because the stabilization of both HIFα proteins after treatment by IOX2 (used as a representative selective PHD inhibitor) was more rapid and of greater magnitude when compared with 0.5% O_2_ treatment over a 16-h period (Fig. 2*D*). Notably, the levels of FIH-catalyzed HIF1α CAD hydroxylation under 0.5% O_2_ suggested that FIH activity was partially inhibited by hypoxia under the tested conditions consistent with FIH being more active than the PHDs under hypoxia (27), whereas CAD hydroxylation was not inhibited with IOX2 treatment alone.

Given the central role of HIF in regulating the transcriptional response to physiological hypoxia, we were interested in investigating potential differences between our identified gene clusters (Fig. 2*B*) and their HIF dependence. We used reported HIF1α and HIF2α ChIP-seq and siRNA data (40, 41) to assess the extent to which genes in each cluster were regulated by HIF. Clusters that contained genes up-regulated in hypoxia (*i.e.* clusters 2–4) were significantly enriched for HIF1α and HIF2α binding (Fig. 2*E*). Those specifically up-regulated by hypoxia and DMOG (cluster 3) showed the strongest evidence for direct targeting by HIF (HIF1α overlap = 30%, HIF2α overlap = 22%). Similar to HIF binding, clusters that contained genes up-regulated in hypoxia were significantly enriched for genes down-regulated by HIF1α siRNA treatment in hypoxia (Fig. 2*F, left*). HIF2α siRNA treatment in hypoxia had a smaller effect on the regulation of hypoxia-inducible genes (Fig. 2*F, middle*) where a combination of the two had an effect more comparable with HIF1α siRNA alone (Fig. 2*F, right*). Using gene set enrichment analysis and the reported HIF ChIP-seq data, we observed a robust association between the HIFα binding and the loci of genes up-regulated by either hypoxia, DMOG, or the PHIs (data not shown).

Together, these results suggest that, at least under the tested conditions, DMOG better mimics the transcriptional response to cellular hypoxia than the (tested) selective PHI. This difference may be a result of increased transcriptional activity of HIF1α and HIF2α due to the 'additional' inhibition of FIH by hypoxia and DMOG compared with the selective PHI alone.

##### Combined PHD and FIH Inhibition Is Required for Optimal Induction of a Subset of Hypoxia-inducible Genes

**FIGURE 3.**
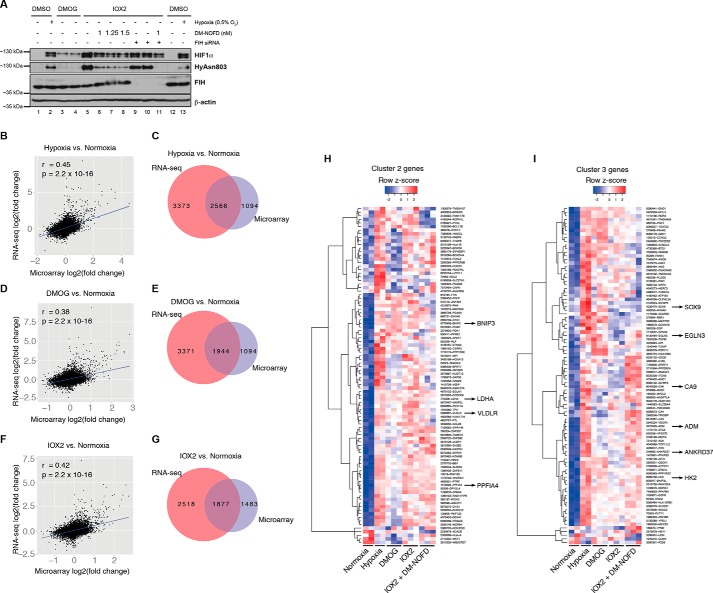
**Hypoxia-regulated genes in response to combined PHD and FIH inhibition.** *A,* immunoblots showing the inhibition of HIF1α asparaginyl hydroxylation using either a small molecule FIH inhibitor (DM-NOFD), siRNA-mediated FIH knockdown, or both in MCF-7 cells. *B* and *C,* correlation of fold changes observed in hypoxia *versus* normoxia between RNA-seq and microarray analyses and the corresponding overlap of genes identified as being differentially expressed in each analysis (FDR < 0.05). *D* and *E,* correlation of fold changes observed in DMOG *versus* normoxia between RNA-seq and microarray analyses and the corresponding overlap of genes identified as being differentially expressed in each analysis (FDR < 0.05). *F* and *G,* correlation of fold changes observed in IOX2 *versus* normoxia between RNA-seq and microarray analyses and the corresponding overlap of genes called as differentially expressed in each analysis (FDR < 0.05). *H,* transcriptional profile of genes in the microarray analysis that were assigned to cluster 2 using RNA-seq data, *i.e.* genes up-regulated in all conditions. Labeled are those genes that were verified using qPCR. *I,* transcriptional profile of genes in the microarray analysis that were assigned to cluster 3 using RNA-seq data, *i.e.* genes up-regulated predominantly by DMOG and hypoxia but not PHI. The genes that were verified using qRT-PCR are labeled. *DMSO,* dimethyl sulfoxide; *l.e.,* long exposure.

Given the greater inhibition of FIH by 0.5% O_2_ and DMOG than by the PHIs (Figs. 1*D* and 2*D*), we proposed that FIH inhibition could explain the differences in transcriptional regulation by hypoxia/DMOG *versus* the PHI. To test the role of FIH, we used short interfering RNA (siRNA) to down-regulate FIH production, as well as the dimethyl ester of a selective FIH small molecule inhibitor, *N*-oxalyl-d-phenylalanine (DM-NOFD (42)) to inhibit FIH activity. NOFD showed lack of *in vitro* inhibitory activities against a panel of Jumonji-C containing proteins KDM3A, KDM4E, and KDM6B (IC_50_ values > 20 μm, data not shown). We found that in MCF-7 cells, FIH siRNA treatment did not completely block HIF1α CAD hydroxylation despite reduction of FIH to below the limit of detection at the protein level (Fig. 3*A*) and >80% reduction at the mRNA level (data not shown). Thus, the residual FIH, not detected by Western blotting, is likely able to hydroxylate the stabilized HIF1α proteins over time. Inhibition of HIF1α CAD hydroxylation was more efficiently achieved by the use of DM-NOFD, which reduced HIF1α CAD hydroxylation to the level comparable with that observed under 0.5% O_2_ (Fig. 3*A*). Complete ablation of FIH activity was achieved by combined DM-NOFD and FIH siRNA treatment (Fig. 3*A*).

**FIGURE 4.**
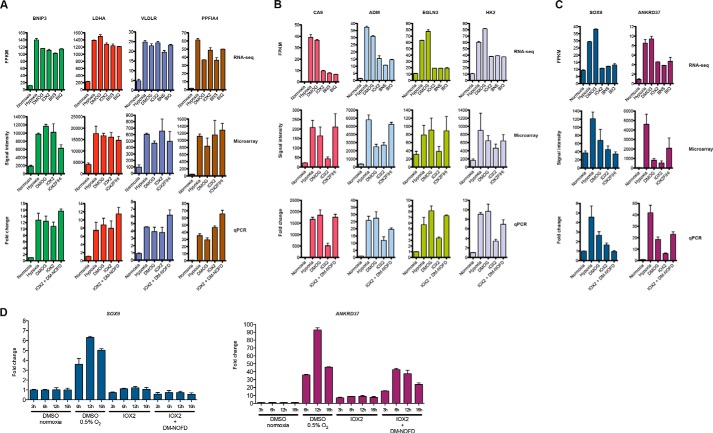
**Hypoxia up-regulated genes have different dependences on the PHDs and FIH.** *A,* RNA-seq, microarray, and qRT-PCR analyses showing an exemplary subset of hypoxia-induced genes (*BNIP3*, *LDHA*, *AK4*, and *PPFIA4*) that are substantially induced by DMOG and the PHIs. *B,* RNA-seq, microarray, and qRT-PCR analyses reveal that a subset of hypoxia-induced genes (*CA9*, *ADM*, *EGLN3*, and *HK2*) with comparable induction by DMOG but not by the selective PHI. Simultaneous use of a PHI and an FIH inhibitor/siRNA induce these genes to a level comparable with that observed in hypoxia. *C,* RNA-seq, microarray, and qRT-PCR analyses reveal that a subset of hypoxia-induced genes (*SOX9* and *ANKRD37*) that are not induced or induced at a lower level by the simultaneous inhibition of the PHDs and FIH. *D,* qRT-PCR analyses showing the time-dependent induction of *SOX9* (*left*) and *ANKRD37* (*right*) with the inhibition of the PHDs or both the PHDs and FIH in comparison with hypoxia. *IOX2FIHi,* 250 μm IOX2 + 1 mm DM-NOFD + 5 nm FIH siRNA; *IOX2* + *DM-NOFD,* 250 μm IOX2 + 1 mm DM-NOFD; *BNIP3,* BCL2/adenovirus E1B 19-kDa protein-interacting protein 3; *LDHA,* l-lactate dehydrogenase A chain; *VLDLR,* very low density lipoprotein receptor; *PPFIA4,* Liprin-α-4; *CA9,* carbonic anhydrase 9; *ADM,* adrenomedullin; *EGLN3,* prolyl hydroxylase domain-containing protein 3; *HK2,* hexokinase-2; *SOX9,* SRY (sex-determining region Y)-box 9; *ANKRD37,* ankyrin repeat domain-containing protein 37. Each *bar plot* represents *n* = 2 per condition.

Using microarrays, we then assessed the ability of dual inhibition of the PHDs and FIH to modulate hypoxia-regulated genes. Although there were some variations between the RNA-seq and microarray analysis for hypoxia, DMOG, and IOX2 conditions (Fig. 3, *B–I*), we identified (and verified by qRT-PCR) candidate gene as follows: (i) those induced by PHD inhibition to levels comparable with those observed in hypoxia without FIH inhibition (*e.g. BNIP3*, *LDHA*, *VLDLR,* and *PPFIA4*; Figs. 3*H* and 4*A*); (ii) those that required both PHD inhibition and additional FIH inhibition for complete induction (*e.g. CA9*, *ADM*, *EGLN3,* and *HK2*; Figs. 3*I* and 4*B*); and (iii) those that could not be induced to levels comparable with hypoxia regardless of PHD and FIH inhibition (*e.g. SOX9* and *ANKRD37*; Figs. 3*I* and 4*C*). Taken together, these results indicate that different genes up-regulated by hypoxia have different requirements for inhibition of the PHDs and FIH for detectable transcriptional activation.

##### Some Hypoxia-inducible Genes Require More than Inhibition of the PHDs and FIH for Transcriptional Activation in MCF-7 Cells

From the gene expression profiling studies, we observed that *SOX9* and *ANKRD37* can be up-regulated by hypoxia (and to a certain degree by DMOG), but they are not optimally up-regulated by both PHD and FIH inhibition. Given that to this point our investigations had mostly focused on a single time point (16 h), we investigated the possibility that these genes may be induced to a level comparable with those in hypoxia at an earlier time point (*i.e.* less than 16 h). qRT-PCR analyses reveal that *SOX9* remained unaffected by IOX2 treatment or combined IOX2 and DM-NOFD treatment over a period of up to 16 h (Fig. 4*D*, *left*). Similarly, although induction of *ANKRD37* gene expression was observed with IOX2 (and combined IOX2 and DM-NOFD) treatment, it did not reach the level of induction observed under hypoxia over a period of up to 16 h (Fig. 4*D*, *right*), consistent with the previous observations (Fig. 4*C*). These results demonstrate that in cultured MCF-7 cells, the incomplete up-regulation of a subset of hypoxia up-regulated genes (such as *SOX9* and *ANKRD37*) by combined PHD and FIH inhibition is not due to time-dependent effects.

##### Requirements for PHDs and FIH for Induction of Hypoxia Up-regulated Genes Are Cell Type-dependent

**FIGURE 5.**
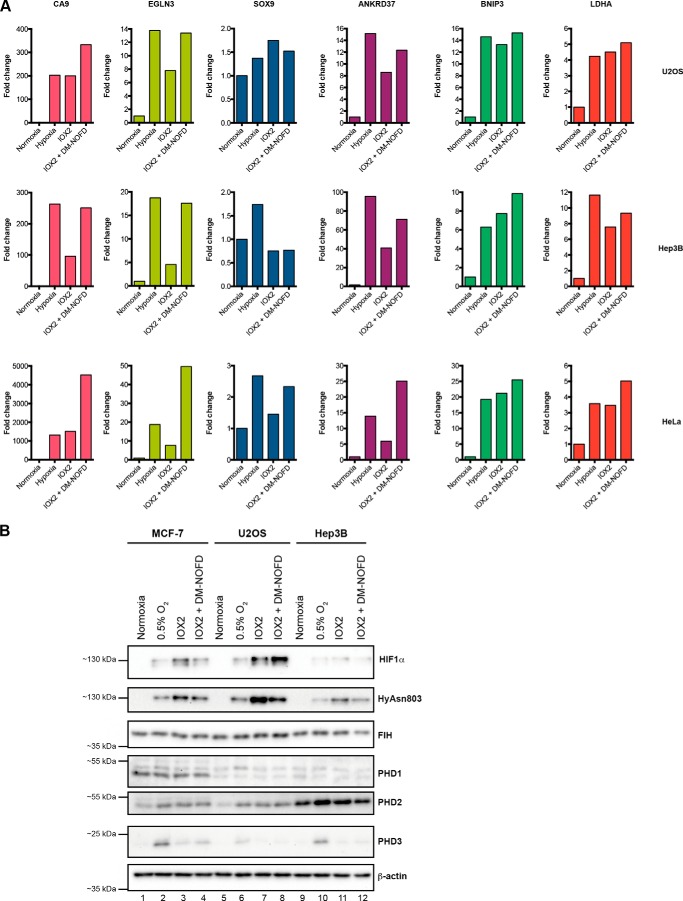
**Dependence of hypoxia up-regulated genes on the PHDs and FIH is context-dependent.** *A,* comparison of the induction of selected hypoxia up-regulated genes in U2OS, Hep3B, and HeLa cells with the inhibition of the PHDs, or both the PHDs and FIH in comparison with hypoxia. Note the differences in results for some of the same genes in different cell types. Data shown are representative of three independent experiments. *B,* immunoblots showing the different levels of HIF1α, HyAsn803, FIH, PHD1, PHD2, and PHD3 under normoxia, hypoxia (0.5% O_2_), or with the treatment of HIF hydroxylase inhibitors in MCF-7, U2OS, and Hep3B cells. *IOX2* + *DM-NOFD,* 250 μm IOX2 + 1 mm DM-NOFD.

To explore whether hypoxia up-regulated genes are differentially regulated by the PHDs and FIH in a similar manner across different cell types, we then studied the effect of IOX2 (*i.e.* a selective PHD inhibitor) and combined IOX2 and DM-NOFD treatment (*i.e.* combined PHD and FIH inhibition) on selected genes in U2OS, Hep3B, and HeLa cells (Fig. 5*A*). *EGLN3* induction in these cell lines requires the inhibition of both the PHDs and FIH to be induced to at least the level seen under hypoxia, as we previously observed in MCF-7 cells. This is also the case for *CA9* induction in Hep3B, but not in U2OS and HeLa cells, whereby the induction of *CA9* by IOX2 alone is comparable with that by hypoxia (although the inhibition of FIH enhances IOX2-mediated induction further). Genes previously observed in MCF-7 cells to be “fully” (relative to hypoxia) induced by IOX2 alone (such as *BNIP3* and *LDHA*) were consistently induced by IOX2 to levels comparable with hypoxia in all the tested cell lines. *SOX9*, a hypoxia-induced gene shown to be non-responsive to the inhibition of PHDs and FIH in MCF-7 cells, is not substantially induced by hypoxia in U2OS and Hep3B cells (fold-change <2), but is hypoxia-induced and responsive to PHD and FIH inhibition in HeLa cells. *ANKRD37*, another hypoxia-induced gene that is up-regulated by IOX2 and DM-NOFD treatment in MCF-7 cells albeit at levels lower than those in hypoxia, can be up-regulated in U2OS, Hep3B, and HeLa cells to levels higher or similar to hypoxia treatment.

## Discussion

Given the links between the HIF-mediated oxygen-sensing and response system and multiple pathological conditions, it is of interest to investigate the extent to which the cellular transcriptional response to hypoxia can be mimicked by small molecule inhibitors. We used high throughput RNA sequencing and microarray gene expression profiling to study the regulation of hypoxia-responsive genes and their dependence on PHD and FIH inhibition by selective small molecule inhibitors. Although genome-wide expression profiling to compare the effects of DMOG to hypoxia has previously been carried out on the HIF system (43), to our knowledge this is the first report of genome-wide expression profiling using selective small molecule inhibitors of the HIF hydroxylases. These results provide insights into the effects of PHI on HIF target gene expression *in vivo*, which may be useful in terms of interpreting the physiological effects of PHI presently in trials for the treatment of anemia (2, 21–23). More generally, the results suggest that the use of small molecules targeting more than one regulatory element to control the activity of pleiotropic transcription factors has considerable potential.

The results reveal that inhibition of the PHDs alone using selective compounds is capable of significantly up-regulating a substantial subset of hypoxia-regulated genes. Notably, however, at least in the studied cells types, the overall transcriptional response to hypoxia is better mimicked by the broad-spectrum 2OG dioxygenase inhibitor DMOG, which likely inhibits multiple types of 2OG oxygenases (32, 33), than by the more selective PHI.

The degree of induction of specific genes varies across the different cell types in response to hypoxia and the different inhibitors (Fig. 5). Although many factors at the post-transcriptional level are potentially involved, this observation may in part reflect the differing levels of HIF proteins and/or the HIF hydroxylases (FIH and PHD1–3) in the different cell lines (Fig. 5*B*). The levels of some of the components of the HIF system also likely vary over the time scales of the analyses; PHD2 and in particular PHD3 are strongly up-regulated by hypoxia/HIF (5). Other factors that may affect expression levels of HIF target genes include variations in the cellular localization dynamics of the HIF isoforms (44) and variations in the levels of other 2OG oxygenases, including the Jumonji C (JmjC) domain-containing histone *N*-methyl lysine demethylases, some of which are regulated by hypoxia/HIF (20, 45, 46). It is also important to note that the PHIs are likely to have different levels of selectivity for the PHD isoforms in cells and may, to varying extent, inhibit other members of the 2OG dioxygenase family (*e.g.* BIQ has been reported to inhibit the fat mass and obesity protein FTO *in vitro* (47)).

There may also be differences in the precise mode of action of PHIs that could affect their activities, *e.g.* some inhibitors may compete with HIFα at the PHD active site and others not (35). Nevertheless, despite the differences in their structures, all three of the selective PHD inhibitors used in this study showed comparable effects on gene expression profiles, implying that there is a subset of hypoxia-responsive genes that can be regulated principally through the inhibition of the PHDs, even in the presence of fully active FIH.

Analyses of 19 gene expression datasets from 14 different cell lines have revealed a relatively small set of genes that are up-regulated consistently and substantially by hypoxia or hypoxia mimetics, consistent with the proposal of a core set of hypoxically up-regulated genes both in different human cell types (24) and in other animals (48). Our overall results are consistent with an important but variable and context-dependent role for FIH in the regulation of HIF target gene expression, *i.e.* the up-regulation of some HIF target genes is substantially more dependent on inhibition of FIH-catalyzed hydroxylation than others. Indeed, previous PHD and FIH silencing studies by RNA interference have shown that hypoxia up-regulated genes have different requirements for the PHDs and FIH to be transcriptionally activated and may reflect the differential regulation of genes across an oxygen gradient (28). This dependence is context-dependent and may reflect differences in the levels of the PHDs, FIH, and HIFα isoforms (and other factors as described above) in different cell types relating to their biological roles in regulating the hypoxic response under different oxygen tensions *in vivo* (27, 31, 38).

At present, the reasons for the differences in the variable extents of the involvement of FIH in HIF target gene expression are largely unknown. HIFα CTAD hydroxylation disrupts the interaction between the CBP/p300 cysteine/histidine-rich 1 (CH1) domains and HIFα CTAD (30). However, HIFα has another site of interaction with the CBP/p300 cysteine/histidine-rich 3 (CH3) domain, *i.e.* via its N-terminal transactivation domain (49), which may influence the extent of FIH involvement in HIF target gene expression. CBP/p300 are transcriptionally activating proteins in part because they contain histone lysine *N*-acetyltransferase and bromodomain domains (50); one possibility is that in the case of some HIF target genes the corresponding histone lysine *N*-acetylation is more limiting for transcriptional up-regulation than is a decrease in FIH activity. The results also clearly imply that in the cases of some genes factors other than PHD/FIH catalysis can limit expression.

An important finding arising from the results is that at least in MCF-7 cells certain genes that are strongly up-regulated in hypoxia cannot be similarly induced by the inhibition of both the PHDs and FIH, for example *SOX9* and *ANKRD37* (Fig. 4, *C* and *D*). Both of these genes have been previously described as HIF target genes (51, 52) and are reported to contain HIF1α- and/or HIF2α-binding sites within the vicinity of their gene loci in the same cell line (MCF-7) used in our studies (40). We demonstrated that the reduced induction or lack of induction by PHD inhibitor and FIH inhibitor in our studies in MCF-7 cells is not due to temporal effects of the inhibitors, as judged by the levels of HIFα, HIF1α CAD hydroxylation, and the induction of the genes across different time points. These observations thus point toward another form of oxygen-dependent transcriptional regulation via an additional factor(s), potentially including 2OG dioxygenases, that may be HIF-dependent or -independent, as indicated by the observation that they are induced by the broad-spectrum 2OG dioxygenase inhibitor DMOG. Such regulation may be direct, *e.g.* by oxygen-dependent regulation of histone demethylation (*e.g.* via modulation of JmjC histone *N*-methyl lysine demethylase activity), or indirect. In the latter regard, it is notable that some 2OG dioxygenases are themselves hypoxically regulated, including PHD2 and -3 and some, but not all, of the 2OG-dependent JmjC histone demethylases (20, 45, 46, 53). The use of chemical probes selective for 2OG dioxygenases and/or gene knockdown studies targeting members of the 2OG-dependent dioxygenase family other than the PHDs and FIH may provide insights into how these additional factor(s) play roles in the hypoxia-mediated up-regulation of these genes.

The 2OG-dependent dioxygenase enzymes all require oxygen to function; however, other than the HIF hydroxylases, there is no evidence that they play direct roles as hypoxia sensors in animal cells. A recent *in vitro* study reports that, like PHD2, a human histone demethylase KDM4E (which is also a member of the 2OG oxygenase) reacts slowly with oxygen (54), a proposed characteristic of hypoxia sensors that has been observed with PHD2 (55) and to much lesser extent with FIH (56). Thus, there is at least potential for the JmjC histone demethylases and other 2OG dioxygenases to act as hypoxia sensors (53, 56). It is also likely that 2OG dioxygenases, including the JmjC histone demethylases, along with multiple other factors, play roles in determining the set of HIF target genes that are hypoxically regulated. It should be noted that demonstration of the oxygen dependence of in cell hydroxylation (*e.g.* as occurs for HIF hydroxylation) is substantially easier than demethylation, because “simple” post-translational hydroxylation does not require a prior post-translational modification as does demethylation. Furthermore, hydroxylation is either present or absent on a given amino acid, whereas a single residue can show different methylation statuses. Along with the complexity of histone modifications (in particular for histone H3), this renders the antibody-based interpretation of changes in hydroxylation modifications substantially easier (at least in our experience) than demethylation (57). Our analyses of reported H3K4me3 ChIP-seq dataset in MCF-7 (41), however, did not reveal any identifiable difference between hypoxia up-regulated genes that are non-responsive to PHD and FIH inhibition (such as *SOX9*) and genes that are responsive (such as *CA9* or *BNIP3*) (data not shown). Hence, a detailed study of the histone methylation status (other than H3K4me3) at the loci of hypoxia-induced genes, which are non-responsive to PHD and FIH inhibition, is of interest with respect to identifying dioxygenases other than the HIF hydroxylases involved in hypoxic sensing, but this is beyond the scope of the current investigation.

Overall, our studies indicate that there are minimally three subsets of hypoxia up-regulated HIF target genes based on their requirement for PHDs and/or FIH inhibition in a context-dependent manner, *i.e.* (i) those apparently requiring only the inhibition of the PHDs for hypoxic up-regulation, (ii) those requiring the inhibition of the PHDs and FIH for hypoxic up-regulation, and (iii) those that are not substantially, or only partially, induced by the inhibition of both the PHDs and FIH in the hypoxia response. In the case of the latter genes, there is the possibility that other 2OG dioxygenases are involved in their transcriptional regulation, potentially in a directly hypoxia-regulated manner. However, there are many other possibilities for regulation of these genes, including by chromatin (histone or DNA modifications) and other post-transcriptional processes that affect RNA levels. Perhaps most notably, the results suggest that the “semi-rational” (*i.e.* based on knowledge of the extensive chemical complexity of the regulation of expression in higher organisms) targeting of combinations of regulatory processes to manipulate the transcription of genes controlled by pleiotropic transcription factors (*e.g.* HIF) will be an interesting avenue for therapeutic benefit.

## Experimental Procedures

### 

#### 

##### Cell Culture and Treatment

Human cell lines (MCF-7, Hep3B, and U2OS) were cultured in Dulbecco's modified Eagle's medium (DMEM and D6546–500ML; Sigma) each supplemented with 10% fetal bovine serum (F7524–500ML; Sigma), 2 mm l-glutamine (G7513–100ML; Sigma), 50 units/ml penicillin, and 50 μg/ml streptomycin (P0781-100ML; Sigma). The MCF-7 cell line was from the American Type Culture Collection (ATCC); the Hep3B cell line was from the European Collection of Cell Cultures (ECACC) (12). The U2OS cell line was a gift from S. Galey (ICRF Clare Hall Laboratories, United Kingdom). Cells were treated either with DMSO (control) or compounds (dissolved in DMSO) and added directly into the cell culture medium to the desired final concentration as described previously (27, 35). For hypoxia (0.5% O_2_) treatment, cells were seeded at least 24 h prior to being incubated for 16 h in an InvivO2 400 hypoxic workstation (Ruskin Technologies, Bridgend, UK).

##### Immunoblotting

Cell extracts were prepared using urea/SDS buffer (6.7 m urea, 10 mm Tris-HCl (pH 6.8), 10% glycerol, and 1% SDS) and processed for immunoblotting as previously described (27). The following primary antibodies were used for immunoblotting: mouse monoclonal HIF1α antibody clone 54 (610958, BD Transduction Laboratories); mouse monoclonal HIF2α antibody clone 190b (58); mouse monoclonal HIF1α hydroxy-Asn803 antibody (a kind gift from Dr. M. K. Lee, Republic of Korea (59)); rabbit polyclonal PHD1 antibody (38); mouse monoclonal PHD2 antibody clone 76a (38); mouse monoclonal PHD3 antibody clone 188e (38); mouse monoclonal FIH antibody clone 162c (31); and β-actin/HRP (clone AC15, Abcam). HRP-conjugated goat polyclonal anti-mouse IgGs (P0447, Dako) were used as secondary antibodies.

##### RNA Preparation

Cells were harvested, and total RNA was prepared using mirVanaTM miRNA isolation kit (AM1560; Life Technologies, Inc.) according to the manufacturer's protocol. Genomic DNA was removed from RNA samples using TURBO DNA-free^TM^ Kit (AM1907; Life Technologies, Inc.) according to the manufacturer's protocol.

##### RNA Seq Library Preparation and Sequencing

Total RNA was subjected to poly(A) selection, and 100 bp of paired-end sequences for the poly(A^+^) fraction were generated on the Illumina HiSeq2000. Library preparation was performed using Magnetic mRNA isolation kit (S1550S; New England Biolabs) followed by NEBNext mRNA sample prep kit for Illumina (E6110; New England Biolabs).

##### RNA-seq Data Analysis

RNA seq reads were aligned to the human reference genome (hg19) using Tophat2 (version 2.0.10). An average of 92.9 (range 88.1 to 109.0 m) reads were mapped, representing an average 93.5% (range 92.1 to 94.6%). Quantification over gene models present in Ensembl (build 72) was performed using gtf2table.py from the CGAT toolkit (60), and average exon counts were used for downstream analysis. Differential expression analysis was performed on each condition contrast using DESeq from R/Bioconductor (version 1.10.1), and differentially expressed genes were identified at FDR of <0.05 and fold change >2. Sequence data have been deposited at the EBI ENA with the accession number E-MTAB-4264.

##### Enrichment for HIF Binding and HIF siRNA Gene Sets

To investigate overlap between gene clusters identified in our RNA-seq data and HIF binding and genes regulated by HIF, we used reported genome-wide mapping of HIF-binding sites by CHIP-seq. Nearest coding gene neighbors of HIF1α- and HIF2α-binding sites were obtained from Tables 1 and 2 as reported in Schodel *et al.* (40). Raw RNA-seq data for HIF1α, HIF2α, and HIF1α + HIF2α, along with scrambled siRNA control data, were downloaded from the European Nucleotide Archive (ENA) with accession number E-MTAB-1994, as reported previously (41). These data were processed in the same way as reported here for our primary RNA-seq data sets; differentially expressed genes were identified for each siRNA *versus* the scrambled control. For each cluster and HIF binding and siRNA gene set combination, we assessed the number of overlapping genes. We derived an empirical significance value by generating an expected overlap distribution for each combination by taking a random set of genes of equal length to the cluster gene set and taking the overlap in 1000 samples. We calculated the *p* value as the fraction of times we observed a greater than or equal overlap to the observed cluster *versus* gene set overlap.

##### Microarray

RNA samples were processed by the Oxford Genomics Centre, Wellcome Trust Centre for Human Genetics, Oxford, UK, for quality control analysis, amplification, and hybridization on HumanHT-12 version 4.0 Expression BeadChip (Illumina, San Diego). Microarray analysis was performed using the LIMMA package (61) in R (version 2.15.2). Signal intensities generated using the BeadStudio (Illumina Inc.) software were normalized for between-array differences using quantile normalization and log_2_ transformation. Differentially expressed probes between each condition and normoxia were called using an empirical Bayes procedure implemented in LIMMA. A total of 21,507 probes corresponding to 17,426 unique genes were analyzed. The microarray data are available at the EBI arrayExpress under the accession number E-MTAB-4300.

##### Quantitative Real Time PCR (qRT-PCR)

Total RNA preparations (after genomic DNA removal) were reverse-transcribed to cDNA using the High Capacity cDNA kit (4374966; Life Technologies, Inc.) according to the manufacturer's protocol. SYBR Green-based qRT-PCR was then performed on the synthesized cDNA using Fast SYBR Green Master Mix (4385612, Life Technologies, Inc.) on an Applied Biosystem StepOnePlus thermocycler (Life Technologies, Inc.). β-Actin was used for normalization, and fold change was determined using the ΔΔ*Ct* method. Sequences of primers used are as follows: ACTB_F, GCTGTGCTACGTCGCCCTG, and ACTB_R, GGAGGAGCTGGAAGCAGCC; ADM_F, TTGGCAGATCACTCTCTTAG, and ADM_R, TTCCACTTCTTTCGAAACTC; ANKRD37_F, TGTGTTGCCGTGCTCAGACAGA, and ANKRD37_R, ACCCACGTGACATCAGCACTTC; BNIP3_F, TGAGTCTGGACGGAGTAGCTC, and BNIP3_R, CCCTGTTGGTATCTTGTGGTGT; CA9_F, AAATCGCTGAGGAAGGCTCAGA, and CA9_R, CAGGGCGGTGTAGTCAGAGA; EGLN3_F, CACGAAGTGCAGCCCTCTTA, and EGLN3_R, TTGGCTTCTGCCCTTTCTTCA; HIF1ΑN_F, CTGTGAACTTCTGGTATAAGG, and HIF1ΑN_R, CTCATTATGGCCACTTTCTG; HK2_F, CCCCTGCCACCAGACTAAACTA, and HK2_R, CAAAGTCCCCTCTCCTCTGGAT; LDHA_F, CACCATGATTAAGGGTCTTTAC, and LDHA_R, AGGTCTGAGATTCCATTCTG; PPFIA4_F, CGGCGGCTAAAGAAGAAACAC, and PPFIA4_R, CAGGAGACCACAGTAGGACCAT; SOX9_F, CTCTGGAGACTTCTGAACG, and SOX9_R, AGATGTGCGTCTGCTC; VLDLR_F, GGAACCGGGAGAAAAGCCAAAT, and VLDLR_R, CCCCATCACATTTCCACAACAG.

##### FIH siRNA and Transfection

Subconfluent MCF-7 cells were trypsinized and resuspended in Dulbecco's modified Eagle's medium (DMEM) without antibiotics before being reverse-transfected with a 5 nm final concentration of Silencer^TM^ select pre-designed and validated FIH siRNA (s31197, Life Technologies, Inc.) using Opti-MEM I reduced serum medium (51985-034; Life Technologies, Inc.) and Lipofectamine RNAiMAX (13778150; Life Technologies, Inc.) according to manufacturer's protocol. 48 h following the transfection, MCF-7 cells were incubated under hypoxia (0.5% O_2_) in InvivO2 400 hypoxic workstation (Ruskin Technologies) or subjected to the indicated inhibitor treatment for 16 h.

##### In Vitro Hydroxylation Assays

Inhibition assays for PHD1–3 were performed by MALDI-TOF MS using a Waters® Micromass® MALDI micro MX^TM^ mass spectrometer via a modified version of the reported procedure (62). Dose responses were assessed by incubation of PHD isoforms (1 μm) with increasing inhibitor concentrations (0.03, 0.1, 0.3, 1, 3, 10, 30, and 100 μm) in the presence of Fe(II) (50 μm), 2OG (10 μm), ascorbate (4 mm), and a 19-mer CODD-peptide (10 μm; DLDLEMLAPYIPMDDDFQL-NH_2_) in 50 mm Tris (pH 7.5) at 37 °C. Reactions were quenched with formic acid (1% v/v) at a time point within the linear region of enzymatic activity. Hydroxylation levels were quantified using MassLynx^TM^ version 4.0, and IC_50_ values were determined with GraphPad Prism®. Inhibition assays for JMJD1A (KDM3A), JMJD2E (KDM4E), JMJD3 (KDM6B), and FBXL11 (KDM2A) were carried out as described previously (33).

##### Protein Expression and Purification

PHD1 full-length enzyme with an N-terminal maltose-binding protein tag was expressed in *Escherichia coli* BL21(DE3) cells. The cultures in 2TY medium were grown to *A*_600_ of 0.6–0.8, then induced with 0.5 mm isopropyl β-d-thiogalactopyranoside; growth was continued at 37 °C for 4 h. Cells were lysed by sonication in 20 mm Tris-HCl (pH 7.5), 200 mm NaCl, and the crude tagged PHD1 was purified over an amylose-affinity column according to the manufacturer's protocol for pMAL^TM^ protein fusion and purification system (New England Biolabs).

PHD2(181–426) with an N-terminal His_6_ tag was produced as described (63). Recombinant PHD3 full-length enzyme with an N-terminal thioredoxin-tag and His_6_ tag was produced in *E. coli* BL21(DE3) cells. Cell cultures in 2TY medium were grown to *A*_600_ of 0.6–0.8 and then induced with 0.05 mm isopropyl β-d-thiogalactopyranoside; growth was continued overnight at 18 °C. Cells were lysed by sonication in 20 mm Tris-HCl (pH 7.5), 500 mm NaCl, 5 mm imidazole, and the crude tagged PHD3 was purified via affinity chromatography over a His trap column (as previously reported by Chowdhury *et al.* (63)).

## Author Contributions

M. C. C. designed and performed experiments, analyzed data, prepared figures, and wrote the manuscript. N. E. I. analyzed data and contributed to the preparation of figures and text. J. S. performed experiments and analyzed data. A. T. performed *in vitro* screening assays for NOFD. K. L. performed *in vitro* assays for PHD1–3. D. S, D. R. M., C. W. P., P. J. R., and C. P. P. analyzed data and contributed to the design of experiments. C. J. S. designed experiments, analyzed data, and wrote the manuscript.

## Supplementary Material

Supplemental Data

## Footnotes

6 The abbreviations used are:

HIF: hypoxia-inducible factor
2OG: 2-oxoglutarate
BIQ: bicyclic isoquinoline
BNS: bicyclic naphthalenylsulfonyl
CBP: cAMP-response element-binding protein-binding protein
CODD: C-terminal oxygen-dependent degradation domain
CTAD: C-terminal transactivation domain
DM-NOFD: dimethyl *N*-oxalyl-d-phenylalanine
DMOG: dimethyl *N*-oxalyl glycine
FDR: false discovery rate
FIH: factor-inhibiting HIF
FPKM: fragments per kilobase of transcripts per million mapped reads
HyAsn803: HIF1α hydroxyasparagine-803
NOG: *N*-oxalyl glycine
NODD: N-terminal oxygen-dependent degradation domains
PHD: prolyl hydroxylase domain
PHI: HIF prolyl hydroxylase domain inhibitor
F: forward
R: reverse
qRT: quantitative RT
RNA-seq: RNA sequencing
EPO: erythropoietin.
