# Supplementary material for: Tuning the Transcriptional Response to Hypoxia by Inhibiting Hypoxia-inducible Factor (HIF) Prolyl and Asparaginyl Hydroxylases
Source: J Biol Chem. 2016 Aug 8;291(39):20661–73. doi: 10.1074/jbc.M116.749291 (PMC5034057; doi:10.1074/jbc.M116.749291)
Supplement: Supplemental Data [file supp_291_39_20661__index.html]

Tuning the transcriptional response to hypoxia by inhibiting HIF prolyl- and asparaginyl-hydroxylases — Tuning the Transcriptional Response to Hypoxia by Inhibiting Hypoxia-inducible Factor (HIF) Prolyl and Asparaginyl Hydroxylases — Transcriptional Regulation via HIF Hydroxylase Inhibition — Supplemental Data 

# Tuning the Transcriptional Response to Hypoxia by Inhibiting Hypoxia-inducible Factor (HIF) Prolyl and Asparaginyl Hydroxylases

## Supplemental Data

- Supplemental Data (.xlsx, 141 KB) - 1081 genes identified as being upregulated in hypoxia compared with normoxia (RNA-Seq).
